# Highlighting the compound risk of COVID-19 and environmental pollutants using geospatial technology

**DOI:** 10.1038/s41598-021-87877-6

**Published:** 2021-04-16

**Authors:** Ram Kumar Singh, Martin Drews, Manuel De la Sen, Prashant Kumar Srivastava, Bambang H. Trisasongko, Manoj Kumar, Manish Kumar Pandey, Akash Anand, S. S. Singh, A. K. Pandey, Manmohan Dobriyal, Meenu Rani, Pavan Kumar

**Affiliations:** 1 Department of Natural Resources, TERI School of Advanced Studies, New Delhi, 110070 India; 2 Department of Technology, Management and Economics, Technical University of Denmark, 2800 Kgs. Lyngby, Denmark; 3 Department of Electricity and Electronics, Institute of Research and Development of Processes IIDP, University of the Basque Country, Campus of Leioa, PO Box 48940, Leioa (Bizkaia), Spain; 4 Remote Sensing Laboratory, Institute of Environment and Sustainable Development, Banaras Hindu University, Varanasi, Uttar Pradesh 221005 India; 5 DST-Mahamana Centre of Excellence in Climate Change Research, Institute of Environment and Sustainable Development, Banaras Hindu University, Varanasi, Uttar Pradesh 221005 India; 6 Department of Soil Science and Land Resource and Geospatial Information and Technologies for the Integrative and Intelligent Agriculture (GITIIA), Bogor Agricultural University, Bogor, 16680 Indonesia; 7 GIS Centre, Forest Research Institute (FRI), PO: New Forest, Dehradun, 248006 India; 8 Directorate of Extension Education, Rani Lakshmi Bai Central Agricultural University, Jhansi, 284003 India; 9 College of Horticulture and Forestry, Rani Lakshmi Bai Central Agricultural University, Jhansi, 284003 India; 10 Department of Geography, Kumaun University, Nainital, Uttarakhand 263001 India

**Keywords:** Projection and prediction, Rehabilitation

## Abstract

The new COVID-19 coronavirus disease has emerged as a global threat and not just to human health but also the global economy. Due to the pandemic, most countries affected have therefore imposed periods of full or partial lockdowns to restrict community transmission. This has had the welcome but unexpected side effect that existing levels of atmospheric pollutants, particularly in cities, have temporarily declined. As found by several authors, air quality can inherently exacerbate the risks linked to respiratory diseases, including COVID-19. In this study, we explore patterns of air pollution for ten of the most affected countries in the world, in the context of the 2020 development of the COVID-19 pandemic. We find that the concentrations of some of the principal atmospheric pollutants were temporarily reduced during the extensive lockdowns in the spring. Secondly, we show that the seasonality of the atmospheric pollutants is not significantly affected by these temporary changes, indicating that observed variations in COVID-19 conditions are likely to be linked to air quality. On this background, we confirm that air pollution may be a good predictor for the local and national severity of COVID-19 infections.

## Introduction

The SARS-CoV-2 coronavirus disease (COVID-19) has emerged as a global pandemic and has so far affected more than 220 countries^1–12^. As of 24 November 2020, this includes more than 59.61 million people worldwide with 1.4 million mortalities and 41.24 million recoveries (Source: Johns Hopkins Corona Virus Resource Center, https://coronavirus.jhu.edu/)^13^. Countries in which COVID-19 has had severe consequences, e.g., in terms of the number of infections and mortalities, include USA, India, Brazil, Russia, France, Spain, United Kingdom (UK), Argentina, Colombia and Mexico^13^. For example, in India, hundreds of people are dying every day due to the deadly virus. Further, due to non-pharmaceutical interventions and policies introduced to cope with the pandemic situation, global and national economic activities in both developing and developed countries have been influenced^14–20^.

As the result of lockdowns and disruption of industrial activities, a significant reduction in air pollution has occurred worldwide, especially with respect to the atmospheric concentrations of carbon monoxide (CO), nitrogen dioxide (NO_2_), sulfur dioxide (SO_2_), aerosols and ozone (O_3_). Clean air is essentially linked to human health. Similarly, changes in air quality and/or the natural atmospheric composition due to, e.g., contamination harms a wide range of living biological organisms either directly or indirectly^21–24^. With the rapid growth of metropolitan areas, particularly in developing countries, considerations of air quality are increasing importance and air quality monitoring has become a critical challenge^25,26^.

Local climatic and environmental conditions and variations can play an important role in the transmission of respiratory diseases, including COVID-19, and exacerbate the risk of morbidity and mortality^26–30^. Several recent studies^30–40^ has reported correlations between air pollutants like PM_10_, PM_2.5_, CO, NO_2_ and O_3_ and mortality rates associated with COVID-19. Taking the example of Northern Italy, which features some of the highest levels of air pollution in Europe, this region has been highly affected by COVID-19 with high incidence and mortality rates^41^. Similarly, several Indian megacities, notably Delhi and Mumbai, which are epicenters of the coronavirus disease, count amongst the world’s most polluted cities^40–44^. Local observations confirm that risks from respiratory diseases tend to worsen in the winter when air pollution peaks. In this regard, air pollution as a factor cannot be neglected when considering the transmission and mortality rates related to COVID-19. Hence, the availability of detailed, spatial information on air pollutants may be important planning tools for mitigating the impact of the COVID-19 pandemic.

Remote sensing techniques are essential tools for mapping air pollutants especially CO, NO_2_, SO_2_, aerosols and O_3_. Since these quantities interact dynamically within the atmosphere, wide coverage remote sensing systems with frequent revisits play a key role. One such system is the Moderate Resolution Imaging Spectroradiometer (MODIS), whose sensors have contributed to a large number of studies on atmospheric pollutants^45^. A newer source of remotely sensed (satellite-based) information is the Sentinel-5 Precursor (5P), which allows end-users to obtain information on trace gases such as methane and carbon monoxide on a daily basis^46–48^. As indicated above, air pollution is largely sourced from activities associated with urban environments (including industrial production) and agriculture where, in particular, the former has been extensively investigated^49,50^.

In this paper, we explore the dynamics of different air pollutants and qualitatively highlight potential links with COVID-19 pressures during different phases of the pandemic for ten of the most affected countries in the world: USA, India, Brazil, Russia, France, Spain, Argentina, UK, Colombia and Mexico. In this respect, we essentially extend the study by (Mohammad et al)^51^, who introduce a similar methodology as means of discussing whether COVID-19 is a “blessing in disguise” based on (visual) observations of a single pollutant (NO_2_) with case examples from China, France, Spain, Italy, and USA. Here, we consider five pollutants (CO, NO_2_, SO_2_, aerosols and O_3_) instead of one and, as just mentioned, ten countries, including also countries in South America, Russia and India. One could easily argue (and rightly so) that more sophisticated methods, machine learning, etc. could be used to yield more detailed quantitative results. However, since the intended benefit of our study lies in extended coverage, in the following we chose to adopt an illustrative methodology similar to that of (Mohammad et al)^51^.

Geospatial data was extracted from the European Commission’s Copernicus Earth Observation Satellite Sentinel-5P. The TROPO spheric Monitoring Instrument (TROPOMI) onboard Sentinel-5P senses air quality parameters, i.e., methane, aerosols, carbon monoxide, nitrogen dioxide, and sulphur dioxide in the lowest layer of the atmosphere (troposphere). Spatially distributed information for prominent pollutants, including CO, NO_2_, SO_2_, aerosols and O_3_, were acquired for three time periods, which conceptually corresponded to different seasonal conditions (e.g., winter, summer, autumn), different phases of the COVID-19 pandemic and also to different levels of mitigating measures. Phase-1 (initial spread of COVID-19) covered the period from 25 January to 31 January 2020; Phase-2 (first wave, extensive lockdowns were implemented in many countries) to the period from 25 May to 31 May 2020); and Phase-3 (second wave of the pandemic, less restrictive interventions) to the period from 25 October to 31 October 2020. The observed levels of air pollution are compared with daily COVID-19 data collected by the Johns Hopkins Corona Virus Resource Center (https://coronavirus.jhu.edu/) and the Government of India, (www.mygov.in).

## Methodology

### COVID-19 data

In general, spatially distributed data on COVID-19 transmission in very high resolution, i.e., at sub-national, state, regional or even city scales are not freely available, and so in the following, we consider only aggregated information at the national level. We collected information on cumulative and active infections and deaths from 22 January 2020 to 10 November 2020, in USA, India, Brazil, Russia, France, Spain, Argentina, UK, Colombia and Mexico from the Johns Hopkins Corona Virus Resource Center (https://coronavirus.jhu.edu/) and the Government of India, (www.mygov.in). For the periods 21 May 2020 to 4 June 2020, and 21 October 2020 to 4 November 2020, corresponding to the above-mentioned Phase-2 and Phase-3, we calculated four indicators, which represent the local state of the COVID-19 pandemic in the ten different countries (Table 1). The length of these periods are slightly longer than the periods used for extracting the remotely sensed data to better account for the daily statistical variations in COVID-19 numbers:

- **New actives per day:** the number of total active COVID-19 cases found for the first and last four days in the time window are averaged, then subtracted, and finally the average daily change in the number of active cases is calculated.
- **Mean no. active cases:** the average number of total active COVID-19 cases during the 15 days.
- **New deaths per day**: the number of total deaths due to COVID-19 cases found for the first and last four days in the time window are averaged, then subtracted, and finally the average daily change in the number of deaths due to COVID-19 is calculated.
- **Death % of no. actives:** ‘New deaths per day’ as a fraction of the ‘Mean no. active cases’.

**Table 1:** Indicators of the state of the COVID-19 pandemic in ten selected countries in Phase-2 and Phase-3.

Date	21 May–4 June 2020 (Phase-2)				21 October–4 November 2020 (Phase-3)			
Country	New actives per day	Mean no. active cases	New deaths per day	Death % of no. actives	New actives per day	Mean no. active cases	New deaths per day	Death % of no. actives
USA	− 933	1,120,335	724	0.065	38,354	5,137,198	601	0.012
India	2189	87,248	145	0.166	− 9918	611,118	387	0.063
Brazil	7535	234,560	701	0.299	− 20,217	487,911	288	0.059
Russia	512	227,337	120	0.053	3792	358,719	217	0.061
Colombia	390	17,636	22	0.127	421	71,706	135	0.189
Spain	327	59,780	33	0.055	13,966	948,960	139	0.015
Argentina	348	9737	25	0.256	− 1147	164,598	248	0.151
France	79	90,111	44	0.049	30,680	1,129,328	230	0.020
Mexico	237	14,615	262	1.790	360	151,961	297	0.196
UK	923	215,157	159	0.074	16,102	897,270	179	0.020

The indicators are based on 15-days of recorded data (number of active infections, the total number of deaths) provided by the Johns Hopkins Corona Virus Resource Center (https://coronavirus.jhu.edu/).

As shown in Table 1 only the USA experienced a significant temporary decline in the COVID-19 transmission rate during Phase-2 (Northern Hemisphere Spring), a picture which is reversed in Phase-3. In Phase-3 (Northern Hemisphere Autumn), a significant decline is experienced in India, Brazil, and Argentina, whereas in Colombia and Mexico just north of the equator the daily transmission rate is almost unchanged from late May to late October (even though the total number of active cases grows by up to a factor of 10).

### Sentinel-5P based datasets

Sentinel-5P based datasets, including the Aerosol Absorbing Index (AAI), Carbon Monoxide Column Density, Tropospheric NO_2_ Concentration, Ozone Total Atmospheric Column and Sulphur Dioxide Column Density, were extracted from (https://code.earthengine.google.com/) andused for monitoring the air quality of the ten countries mentioned above. Sentinel-5P uses the TROPOMI instrument, which has a multispectral sensor that records reflectance of wavelengths optimum for measuring the atmospheric concentration of gases at a spatial resolution of 0.01 arc degree. The retrieval of Sentinel-5P data, its pre-processing and map generation werecarried out using the Google Earth Engine^52^, which is a cloud-based platform widely used for processing satellite data.

To illustrate the spatio-temporal variation of different air pollutants, the abovementioned quantities were obtained for three different periods as listed above, corresponding to the initial phase of the COVID-19 pandemic, the mid-2020 situation (first phase of the disease) and a late-2020 situation (second or in some cases even the third phase of the disease):

- Phase-1 (25 January 2020 to 31 January 2020).
- Phase-2 (25 May 2020 to 31 May 2020).
- Phase-3 (25 October 2020 to 31 October 2020).

For comparison, we also extracted comparable data for similar periods in 2019.

### Indicators of air pollution

The *Aerosol Index Product* provided by Sentinel-5P is a qualitative index that measures the presence of aerosols with substantial absorption. Mathematically, the aerosol index can be expressed by:

1 $$ AAI = 100\log_{10} \left( {\frac{{R_{meas} \left( {\lambda_{2} } \right)}}{{R_{meas} \left( {\lambda_{1} } \right)}}} \right) - 100\log_{10} \left( {\frac{{R_{meas} \left( {\lambda_{2} } \right)R_{calc} \left( {\lambda_{1,} A_{LER} \left( {\lambda_{1} } \right)} \right)}}{{R_{meas} \left( {\lambda_{1} } \right)R_{calc} \left( {\lambda_{2,} A_{LER} \left( {\lambda_{2} } \right)} \right)}}} \right) $$

where AAI is the aerosol absorbing index; R_meas_ depicts measured reflectance at wavelengths λ_1_ and λ_2_; R_calc_ describes calculated reflectance from the atmosphere with Rayleigh scattering; A_LER_ is the Lambert equivalent reflectivity, which is the measured reflectance for wavelength λ_2_.

The *Carbon Monoxide Product* is used to estimate the total column that needs to be retrieved—not only for background CO abundance but also for surface reflection. A physics-based retrieval approach was usedto derivethe scattering properties of the observed atmosphere and associated trace gases^53,54^.

Nitrogen dioxide (NO_2_) and nitrogen oxide (NO) referred together as nitrogen oxides are significant trace gases that are the end products of anthropogenic sources as well as natural processes.

Ozone profiles were utilized to monitor the development of stratospheric ozone. Two types of products were used; the first covered the entire atmosphere and was derived in the spectral range of 270–320 nm. The second one covered the tropospheric profile, spanning the spectral range from 300 to 320 nm.

A Slant Column Density (SCD) is derived using the log ratio of the observed UV–visible spectrum, backscattered radiation from the atmosphere and the observed spectrum. The SCD depicts the gas concentration, which is bias-corrected and converted into vertical columns through air mass factors. The vertical columns are evaluated mathematically by

2 $$ Nv = \frac{{N_{s} - N_{s}^{back} }}{M} $$

where N_v_ and N_s_ respectively represent the vertical and slant column density; N_s_^back^ depicts values for background correction, and M is the air mass factor.

## Results and discussion

### COVID-19 pressures

As shown in Table 1, in all of the ten countries the number of active COVID-19 cases increased from late May to late October, ranging from a 50% increase in Russia to a multiplier of 16–17 times in the case of Spain and Argentina. Except for USA and Brazil, the average daily number of deaths due to COVID-19 also increased, indicating a steeper trajectory, although in relative terms the mortality rate of the disease, measured as the number of daily deaths divided by the total number of active infections, in most cases decreased. All of these numbers of course mask out very large (spatial) variations. Hence, transmission rates of COVID-19 and thereby total numbers of infections are high in densely populated urban areas, where also air pollution is generally a major challenge. Five of the ten countries depicted here, which currently ranks amongst those countries in the world most affected by COVID-19: USA, India, Russia, Brazil and UK are also amongst those with the worst quality of air (see below). On this background, it is reasonable to hypothesize that the previously documented relationship^31–40^ between (poor) air quality and the severity of respiratory diseases also affect the risk of morbidities and mortalities related to COVID-19. A formal attribution of this effect is however beyond the scope of the current study.

### Spatiotemporal distribution of air pollutants

Time-series data of AAI, CO, O_3_, SO_2_ and NO_2_ were extracted for the three phases, corresponding to the last week of January (outbreak of COVID-19), May (main lockdown period), and October (normalized situation or partial lockdown). In general, the geographical repartition of CO, O_3_, SO_2_ and NO_2_ values delineate a set of characteristic pollution patterns for the USA, European and Asian countries, which changed between phases.

**Figure 1:**
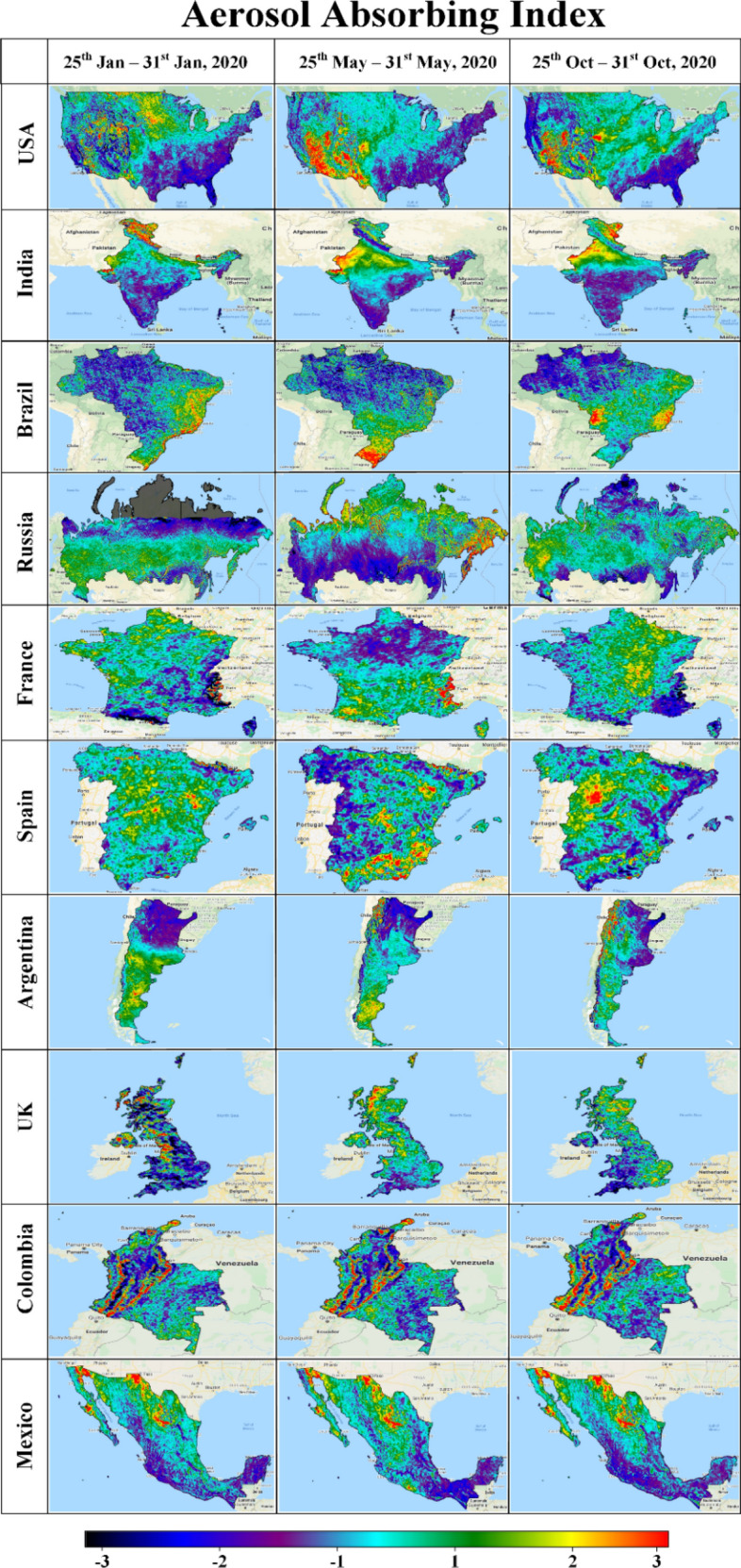
Aerosol absorbing index of the top ten most affected countries^32^.

AAI is a qualitative index indicating the presence of elevated layers of aerosols. Figure 1 indicates significant absorption for all of the countries in this study: USA, India, Brazil, Russia, France, Spain, Argentina, UK, Colombia, and Mexico. Desert dust, biomass burning and volcanic ash plumes are likely to be the main contributors to the AAI and can be derived over clear as well as (partly) cloudy pixels. In Fig. 1, large AAI is generally found for the northern parts and west of the central part of Russia, USA, India, UK, Colombia, Mexico and Spain due to desert dust and anthropogenic pollution. The AAI over the western and eastern coasts of the USA can also be attributed to anthropogenic sources, as those regions are the main industrialized areas in the country. States in the USA dominated by agricultural sectors like the Midwest are generally dominated by lower values of the AAI. Industrial emissions in South America are also assumed to be important contributors to the global aerosol production. Overall, the spatial patterns appear to be similar for all retrievals; while small differences in the retrievals (e.g. varying pixel intensities) may be due to seasonal variations, they could also be explained by other factors including aerosol model assumptions, sensor calibration and cloud screening. Inter-annual variability of smoke intensities in the south of the USA and Brazil regions are closely tied to the drought cycle. Meanwhile, aerosol amounts over India, Brazil, Russia, France, Spain, Argentina, UK, Colombia and Mexico sites are mostly dominated by the smoke generated from fires associated with savanna and forest clearing practices.

Comparing the spatial distribution of the AAI across all three phases (Phase-1 to Phase-3), unchanged (or slightly intensified) patterns were principally found for Mexico and Colombia. This suggests that there has been no significant change in the environmental air quality amid the restrictions installed by the Colombian and Mexican Governments since March 2020. Conversely, in other countries, the imposed restrictions caused by lockdowns (Phase-2) appear to have had a positive impact with respect to aerosols, which would be a favorable condition for COVID-19 mitigation.

**Figure 2:**
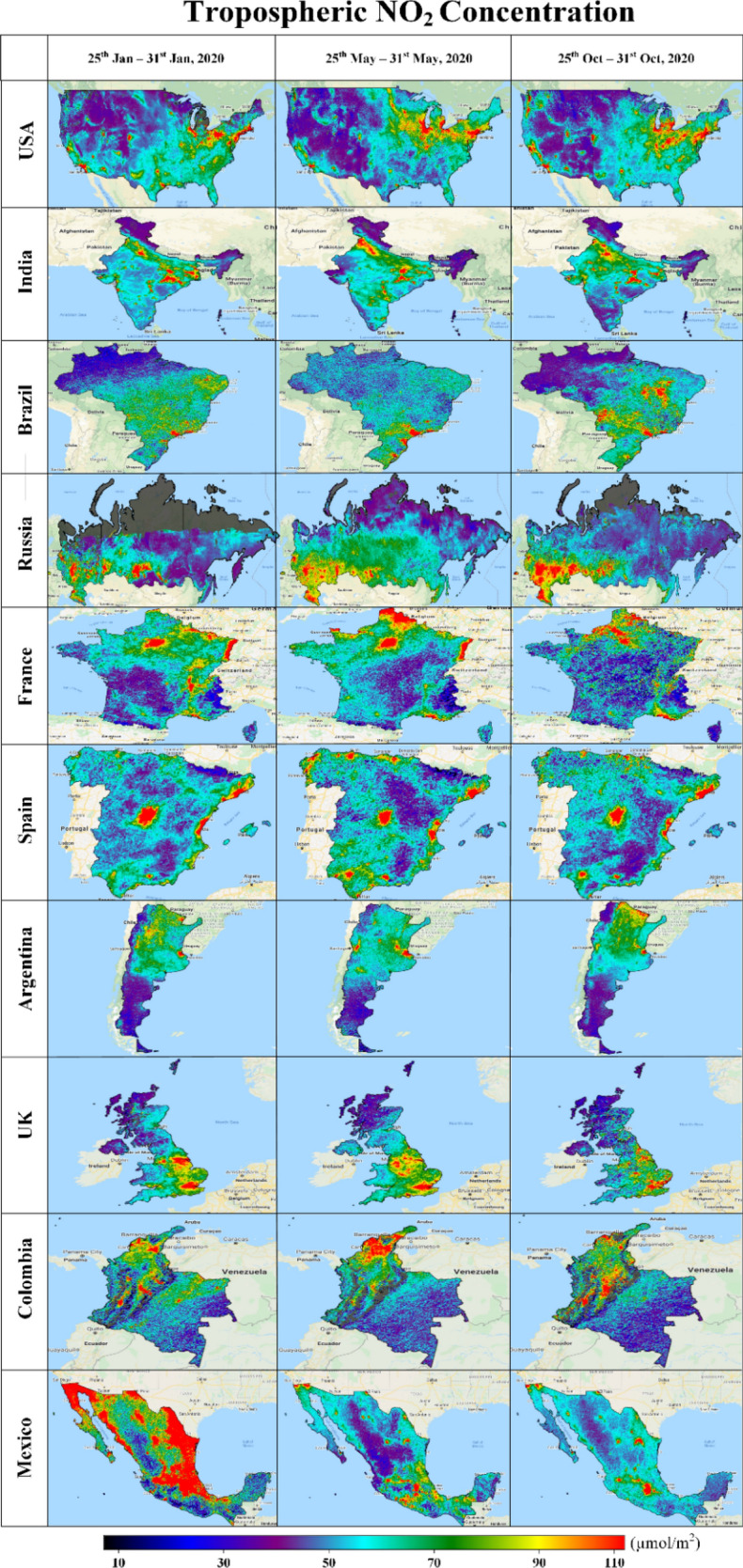
Tropospheric nitrogen dioxide concentration of top ten most affected countries^36^.

**Figure 3:**
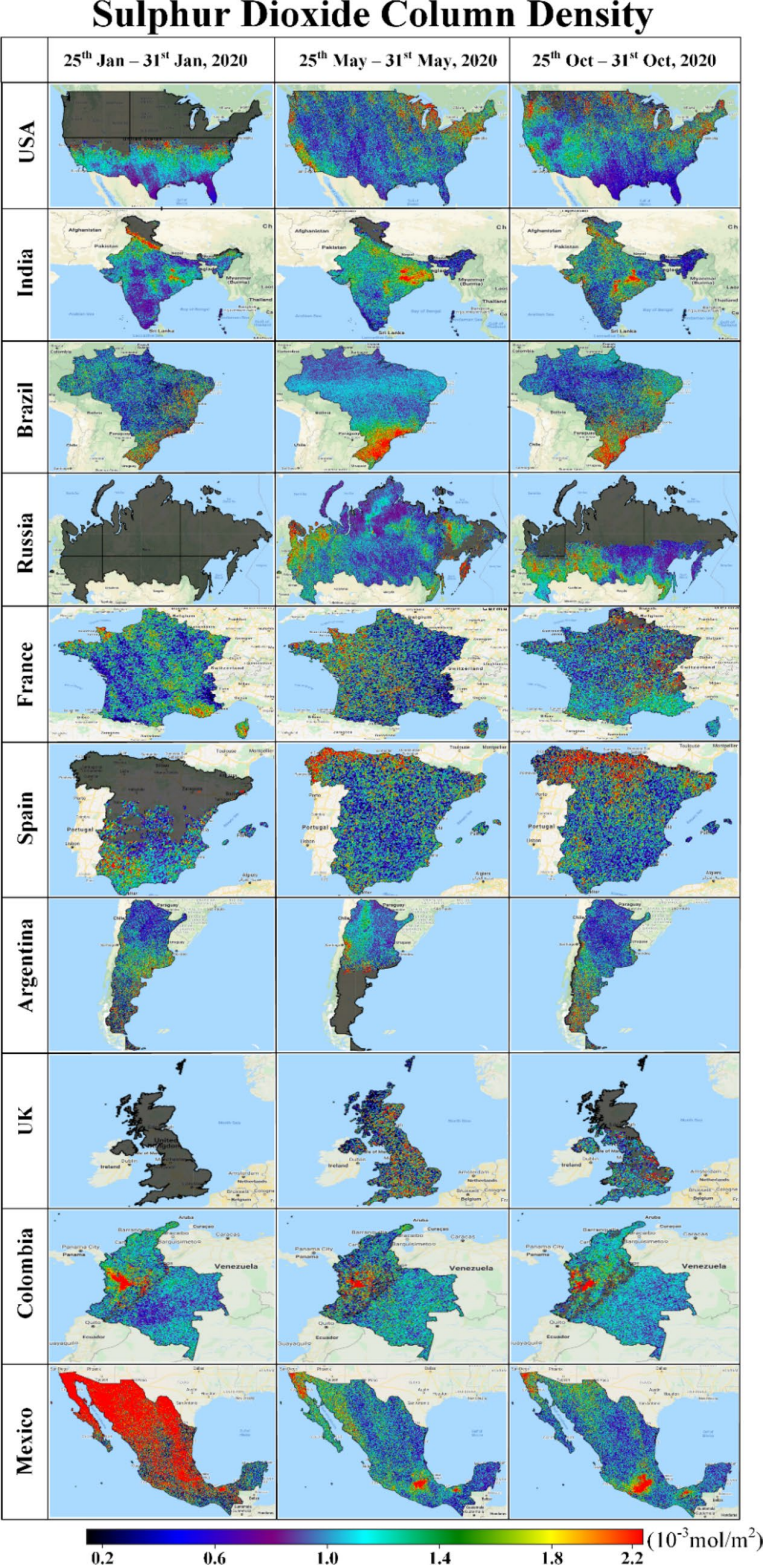
The sulphur dioxide column density of the top ten most affected countries^36^.

Analogous trends are depicted in Figs. 2 and 3 (S1, S2 in the Supplementary Material), indicating improvements in air quality with respect to several of the indicated trace elements, i.e., as a side effect of measures introduced to mitigate the spread of COVID-19 from March to June 2020. For all countries, the level of CO generally decreased though with significant spatial variation (Figure S1). NO_2_ levels also decreased generally in some countries during Phase-2 (Fig. 2). Conversely, e.g., in India, USA and Russia, regional concentrations of NO_2_ and O_3_ (Figure S2) increased significantly, in some case by more than 50% during the “lockdown” Phase-2 as compared to Phase-1.

In terms of nitrogen and sulphur dioxide pollutants (Fig. 3), very significant improvements in air quality are seen in Mexico. At the time of the first reported COVID-19 cases in Mexico (Phase-1), levels of atmospheric nitrogen and sulphur dioxide pollutants were extremely high, especially in the regions of Baja California in the West and the North and Eastern regions, including Coahuila, Nuevo Leon and Tamaulipas. Shortly after the restriction was imposed, NO_2_ and SO_2_ amounts declined, suggesting that recorded morbidities and mortalities due to COVID-19 in Mexico would be less affected by these aspects of air quality.

Comparing the results found for Phase-2 and Phase-3 (Figs. 1, 2, 3, S1, S2), it is evident that the observed reductions in air pollution levels resulting from the (partial) lockdowns were only temporary. Not accounting for seasonal variations, as global and national economic activities resumed, levels of air pollution started to rise again from May to October (e.g., Phase-3).

### Temporal dynamics of CO, O_3_, SO_2_ and NO_2_ between 2019 and 2020

**Figure 4:**
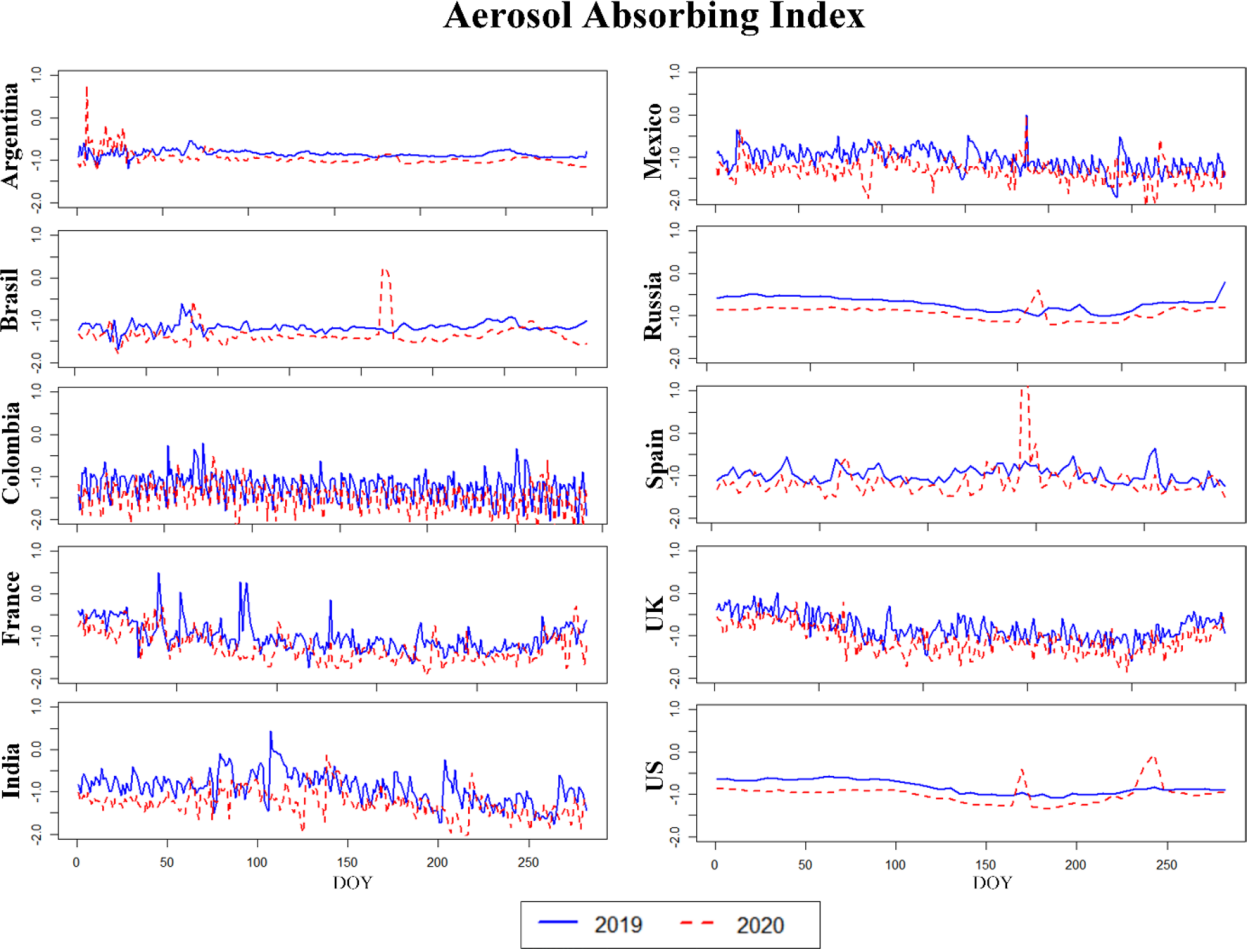
Aerosol absorbing index (AAI) of the countries for the years 2019 and 2020. *DOY* day of the year.

**Figure 5:**
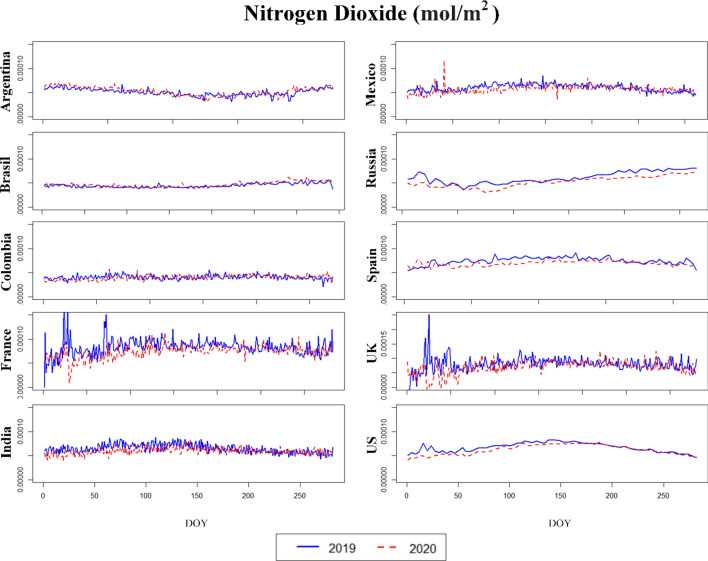
Nitrogen dioxide of the countries for the years 2019 and 2020.

**Figure 6:**
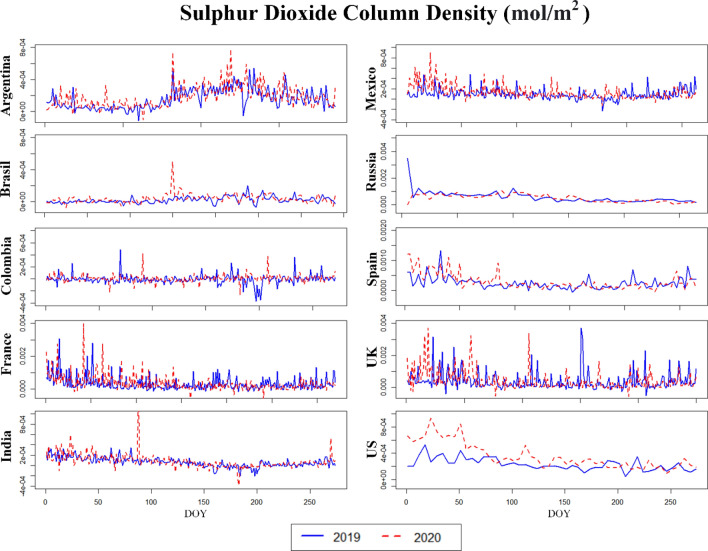
Sulphur dioxide column density of the countries for the years 2019 and 2020. *DOY* day of the year.

Figures 4, 5, 6 (and S3, S4 in the Supplementary Material) illustrate the temporal (daily) dynamics and seasonal variability of the same set of pollutants (aerosols, CO, O_3_, SO_2_ and NO_2_) as depicted on Figs. 1, 2, 3. The blue curves correspond to data from 2019 whereas the red curves indicate data for 2020. The mid-points of the three phases studied above correspond to the following days-of-year (DOY): **28** (28 January 2020), **149** (28 May 2020) and **302** (28 October 2020). Both of the time series (2019, 2020) are truncated after Phase 3 (Figs. 4, 5, 6, S3 and S4).

Assuming 2019 to be a “normal year”, we generally find that the seasonal variations in 2020 are preserved for all variables despite the modifications brought about by the implementation of COVID-19 policies, including lockdowns. For the developing countries Mexico and Colombia, a highly fluctuating AAI is observed both in 2019 and in 2020 (Fig. 4), which is probably not related to COVID-19 but due to some other cause like the frequent biomass burning for energy purposes in the countryside.

As already mentioned, net reductions in CO, O_3_, NO_2_ and SO_2_ were observed for many countries in 2020—particularly when going towards May 2020. These reduction scans are attributed to the complete or partial lockdown of industrial activities and vehicle traffic across the American, Asian and European continents. To exemplify, by population, Europe ranks third among regions of the world with 9.8% of the total population in the world living in Europe (Worldometer’s 2020 statistics). Over 70% of the European population, however, lives in urban areas, explaining the generally high levels of CO, O_3_, SO_2_ and NO_2_ pollution. By the end of May 2020, multiple European cities that previously featured high levels of air pollution (CO, O_3_, NO_2_ and SO_2_) now reported very low pollution levels, indicating improvements in air quality. Comparing 2019 and 2020 (Fig. 5), the tropospheric NO_2_ column number density maintained high values of about 0.0001 mol/m^2^ for the USA, France and UK. In countries like Argentina, Brazil, Colombia, Russia, and Spain the equivalent numbers were found to be lower (closer to below 0.0001 mol/m^2^ in 2019) and improving during the principal 2020 lockdown.

For several major cities in France, Spain, UK, USA and Russia, O_3_ concentrations increased in 2019 compared to previous years (Figure S4). They slightly decreased in 2020 especially during the lockdown (e.g., Phase-2) (0.16 mol/m^2^) (Figure S4). In the Latin American countries, represented by Columbia, Brazil, Argentina and Mexico, O_3_ concentrations were comparably found to be lower (0.12 and 0.14 mol/m^2^ respectively) than the just mentioned countries. This is in line with the fact that many lower-income countries emit significantly lower levels of atmospheric pollutants. Finally, for SO_2_ France and the USA recorded a high level of concentration (~ 0.002 mol/m^2^) (Fig. 6), although we also note that the spatial distribution of SO_2_ pollutants is influenced by local factors in almost all cases.

Several authors have already demonstrated a correlation between air pollution and morbidity and mortality linked to respiratory diseases and in particular to COVID-19. Given the different scope and (coarse) resolution of the COVID-19 data used, it was however not possible to carry out a similar attribution within this study. In a qualitative sense, it is evident though that the relatively moderate health and multi-sectorial impacts suffered in the Spring of 2020 (compared to the present situation worldwide) may have taken advantage of the significantly lower levels of select air pollutants that was a welcome but unexpected side effect of the lockdowns introduced in many countries. For example, this could prospectively help to explain the positive situation in the USA in late May 2020, which only a few months later was turned upside down. Conversely, our results also seem to indicate that seasonal variations alone are insufficient as a means of explaining the potential variation in the risk of, e.g., mortalities due to COVID-19. That said, as already suggested by our analysis, air pollution—especially in cities—tend to reach a high point in the winter season. When combined with the current (and very alarming) growth in the incidence rates of COVID-19 all over the world, this could exacerbate the already very critical situation in many countries, where COVID-19 threatens the capacity of national and local health systems. This could for example be true in developing countries like India, where 21 out of 30 major cities are listed among the most polluted cities in the world.

## Conclusion

In this study, we explore the utility of remotely sensed data as a means of qualitatively explaining the observed developments of the COVID-19 pandemic, and in particular, the varying risk of a deadly outcome. On this background, data from Sentinel-5P was retrieved for 2019 and 2020. For 2020, three conceptual phases of the diseases were investigated: an early stage at the end of January (Phase-1), a stage a least partly overlapping the extensive lockdown, which was demanded in many countries, starting from around March (Phase-2); and finally, a stage in late October, where lockdowns had been relaxed, leading to resumed local and global economic activities (Phase-3).

Using data extracted from the Johns Hopkins Corona Virus Resource Center, we illustrate the temporal development of the novel coronavirus in ten of the most severely affected countries in the world. From Phase-2 to Phase-3, the globally accumulated numbers of COVID-19 infections have increased dramatically with the USA in the less fortunate role of being first on the list. For India, Brazil and Argentina, a decline in the number in active infections is observed, for Columbia and Mexico the numbers are largely unchanged, whereas for the remaining countries (USA, Russia, France, Spain and the UK) the development follows the increasing global trend.

Comparing different indicators of air pollution for 2019 and 2020, despite the anomalous modifications introduced by the lockdowns, seasonal variations were generally found to be unchanged, and indicating that observed variations in COVID-19 conditions are likely to be linked to air quality. Further, the level of most pollutants temporarily declined in Phase-2. On this background, our study confirms that air pollution may be a good predictor for the local and national severity of COVID-19 infections.

## Supplementary Information

Supplementary Information.

## Data Availability

Data on COVID-19 was acquired from the repositories of the Johns Hopkins Corona Virus Resource Center, the Worldometer and the Ministry of Health and Family Welfare, Government of India. These data sources are freely accessible through web-based archives.
