# Supplementary material for: Highlighting the compound risk of COVID-19 and environmental pollutants using geospatial technology
Source: Sci Rep. 2021 Apr 16;11:8363. doi: 10.1038/s41598-021-87877-6 (PMC8052456; doi:10.1038/s41598-021-87877-6)
Supplement: Supplementary file 1 — Supplementary Information. [file 41598_2021_87877_MOESM1_ESM.docx]

**Highlighting the compound risk of COVID-19 and environmental pollutants using geospatial technology**

Ram Kumar Singh, Martin Drews, Manuel De la Sen, Prashant Kumar Srivastava, Bambang H. Trisasongko, Manoj Kumar, Manish Kumar Pandey, Akash Anand, S.S. Singh, A. K. Pandey, Manmohan Dobriyal, Meenu Rani, Pavan Kumar

**Supplementary Information**

Figure S1. Carbon Monoxide Column Density of the top ten most affected countries.

Figure S2. Ozone Total Atmospheric Column of the top ten most affected countries.

Figure S3. Carbon Monoxide of the countries for the years 2019 and 2020.

Figure S4. Ozone Total Atmospheric Column of the countries for the years 2019 and 2020.


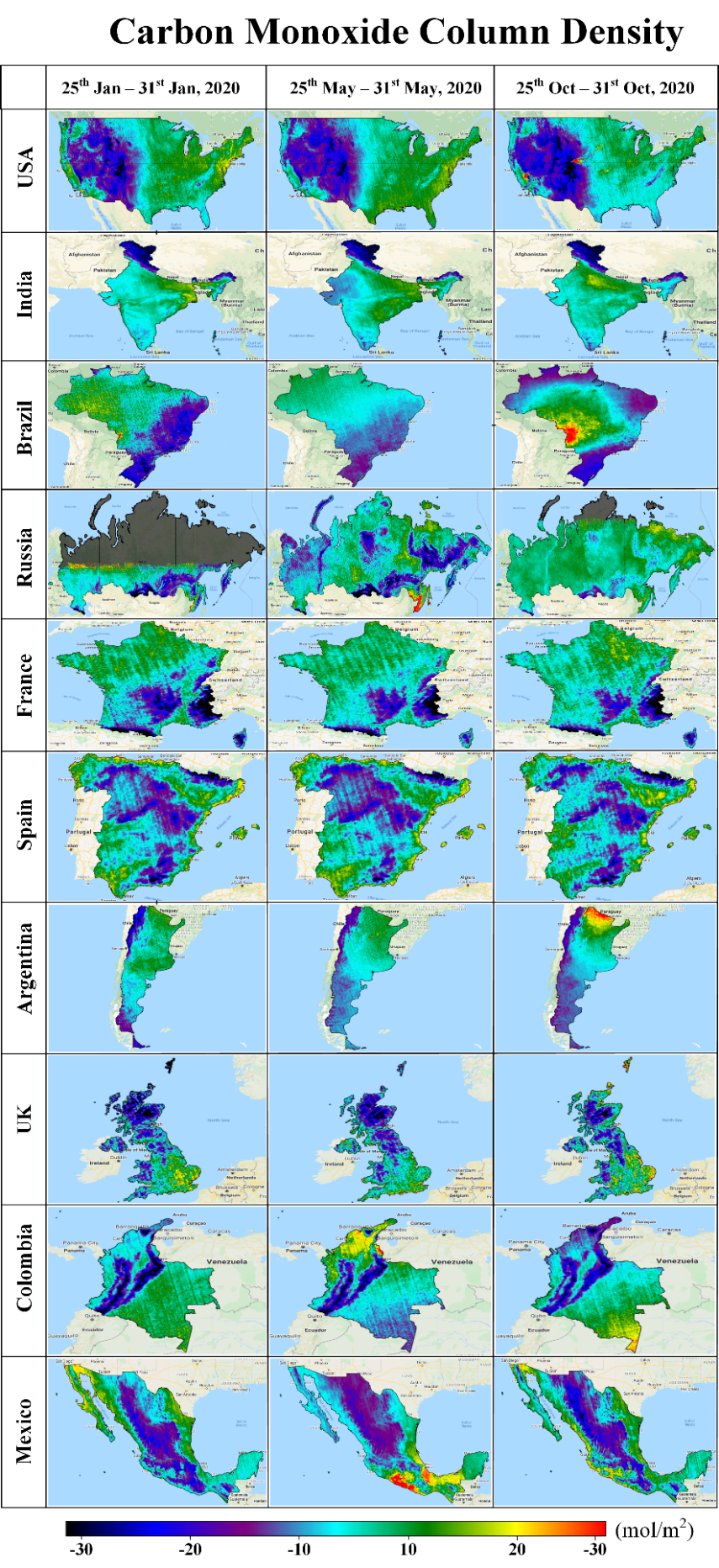


**Figure S1.** Carbon Monoxide Column Density of the top ten most affected countries^33^.


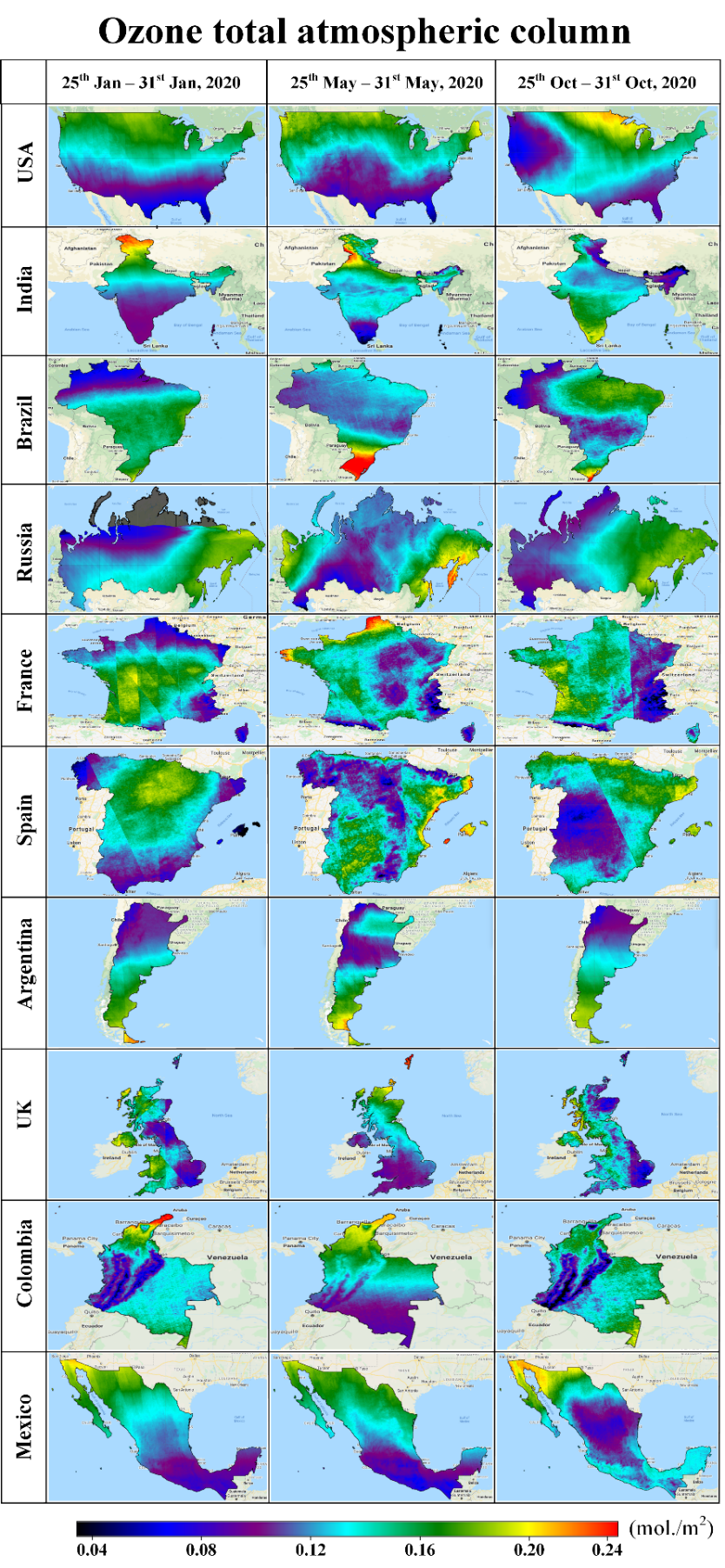


**Figure S2.** Ozone Total Atmospheric Column of the top ten most affected countries^33^.


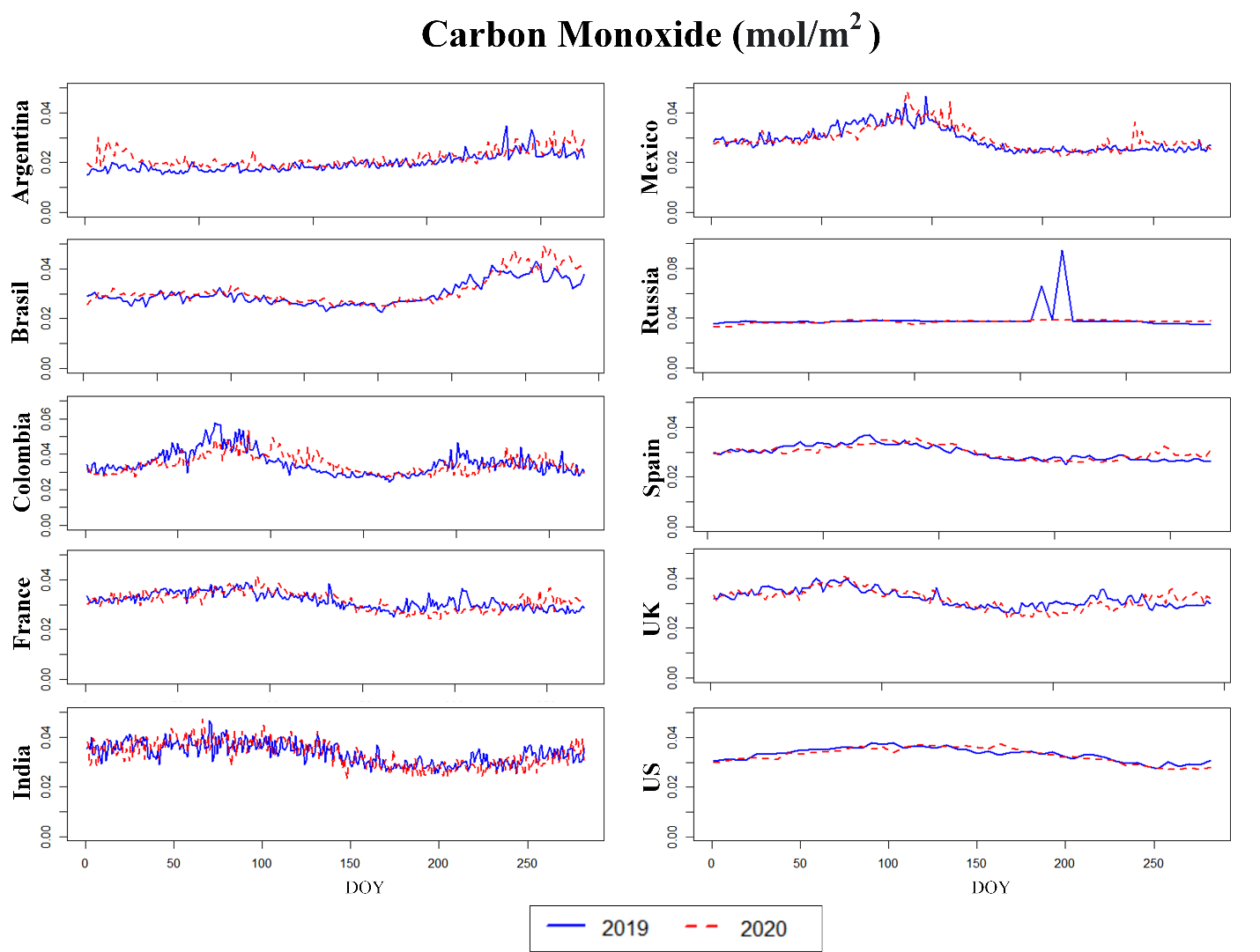


**Figure S3.** Carbon Monoxide of the countries for the years 2019 and 2020.

DOY = day of year.


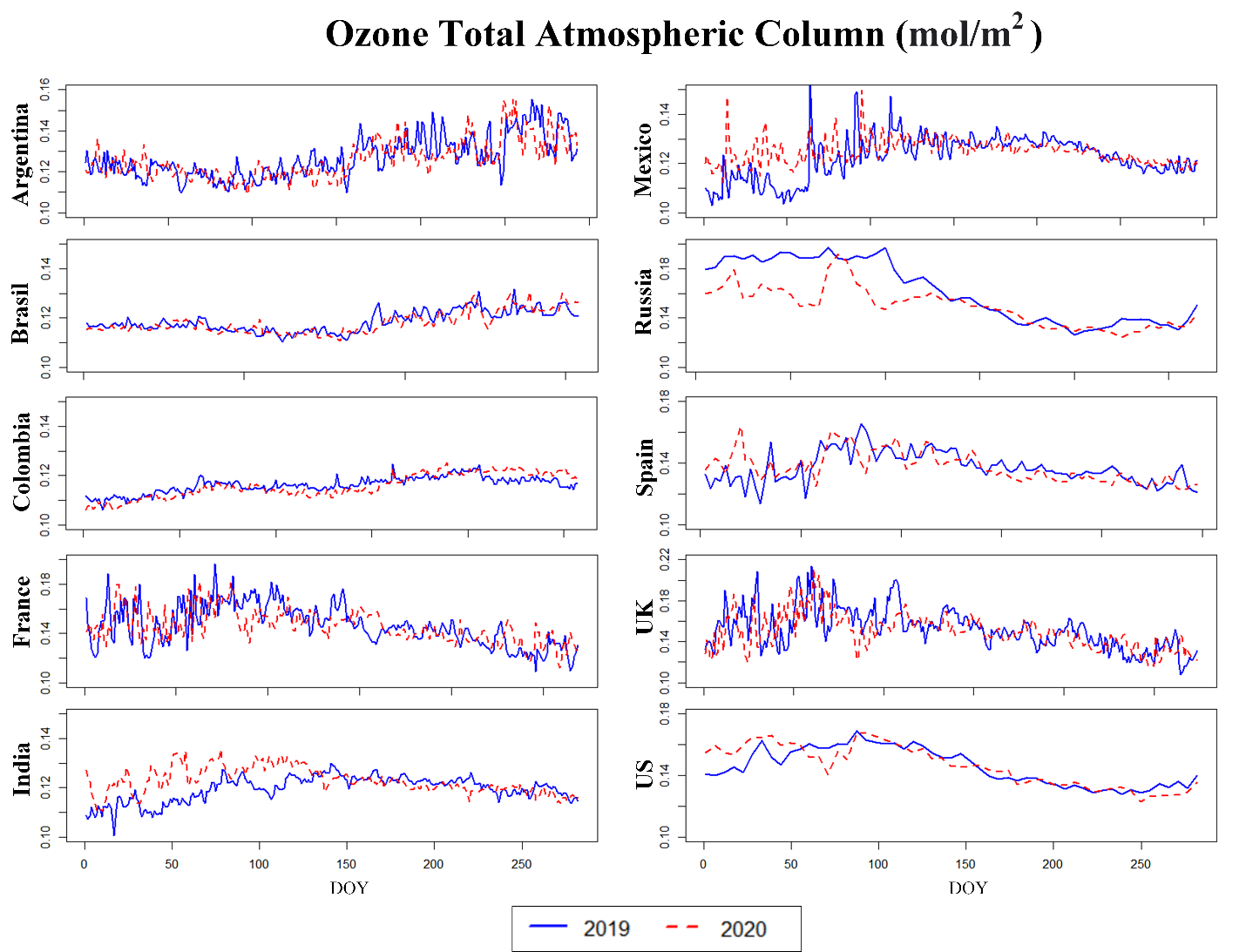


**Figure S4.** Ozone Total Atmospheric Column of the countries for the years 2019 and 2020.

DOY = day of year.
